# Cardiovascular magnetic resonance feature tracking in patients with acute myocarditis and normal ejection fraction: potential for improved diagnosis and prognosis

**DOI:** 10.1186/1532-429X-17-S1-M7

**Published:** 2015-02-03

**Authors:** Mohammed Y Khanji, Mahvesh R Javaid, Saidi A Mohiddin, Redha Boubertakh, Neha Sekhri, Steffen E Petersen

**Affiliations:** 1 Center for Advanced Cardiovascular Imaging and Research, William Harvey Research Institute, Queen Mary University London, London, UK; 2 Cardiology, Barts Health NHS Trust, London, UK

## Background

Myocarditis is the most common finding in patients presenting with chest pain, ECG changes, raised troponin and culprit-free coronary angiograms. Adverse events, such as arrhythmias, heart failure and death can occur in the absence of significant left ventricular (LV) impairment and myocarditis should not be considered benign when systolic function is normal at presentation. We currently do not know how to predict which patients will deteriorate. In addition, poorly understood risks of sudden death and cardiovascular events persist in this group.

We sought to assess changes in cardiovascular magnetic resonance (CMR) feature tracking (FT) parameters between baseline and follow up scan in myocarditis patients with LV ejection fraction (EF) above 55% on admission to see if FT may help predict interval changes in ejection fraction and possibly adverse outcomes.

## Methods

We prospectively followed 33 consecutive patients presenting with chest pain, troponin elevation and unobstructed coronaries. 17 of these had LVEF above 55% and were included in the current analysis. Baseline scans were performed during admission and follow-up within 3 months. Three dimensional peak LV strain, strain-rate and myocardial velocities were measured (TomTec feature tracking software) for both baseline and follow up scans using 2 chamber (2c), 4 chamber (4c) and 3 short axis slices; basal (sAb), mid (sAm) and apical (sAa) using cine SSFP images. Paired t-test was performed to compare interval parameters.

## Results

**Figure 1:**
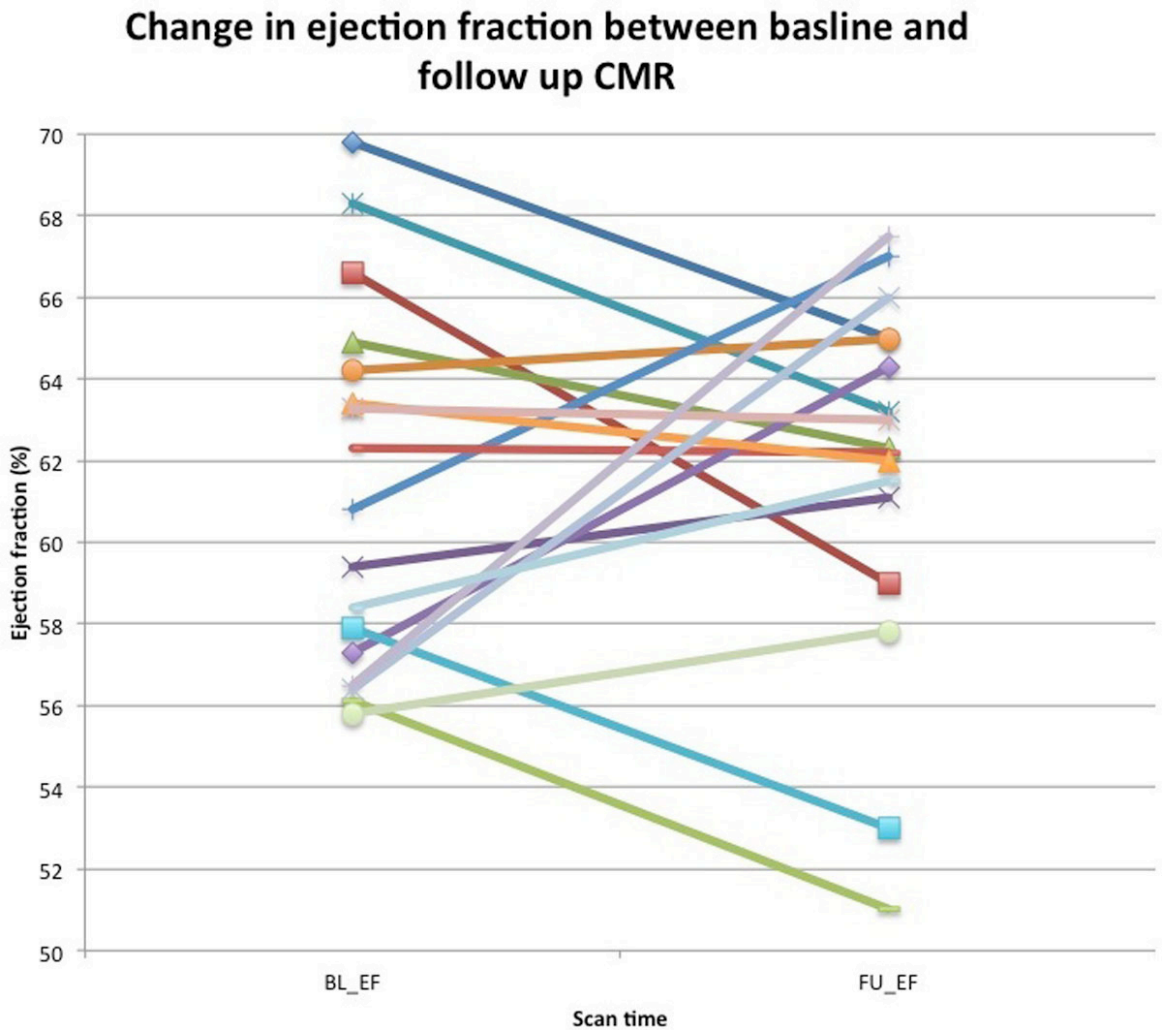
Interval change in left ventricular ejection fraction between baseline (BL) and follow up (FU) CMR.

Mean age was 35.8 years and 12 were male. There was no significant difference in the mean EF between baseline and follow up (61.26% ± 4.5% (±1SD) vs 61.82% ± 4.5%, p=0.304). 2 patients at follow developed mild LV systolic dysfunction (LVEF <55%; figure 1).

**Figure 2:**
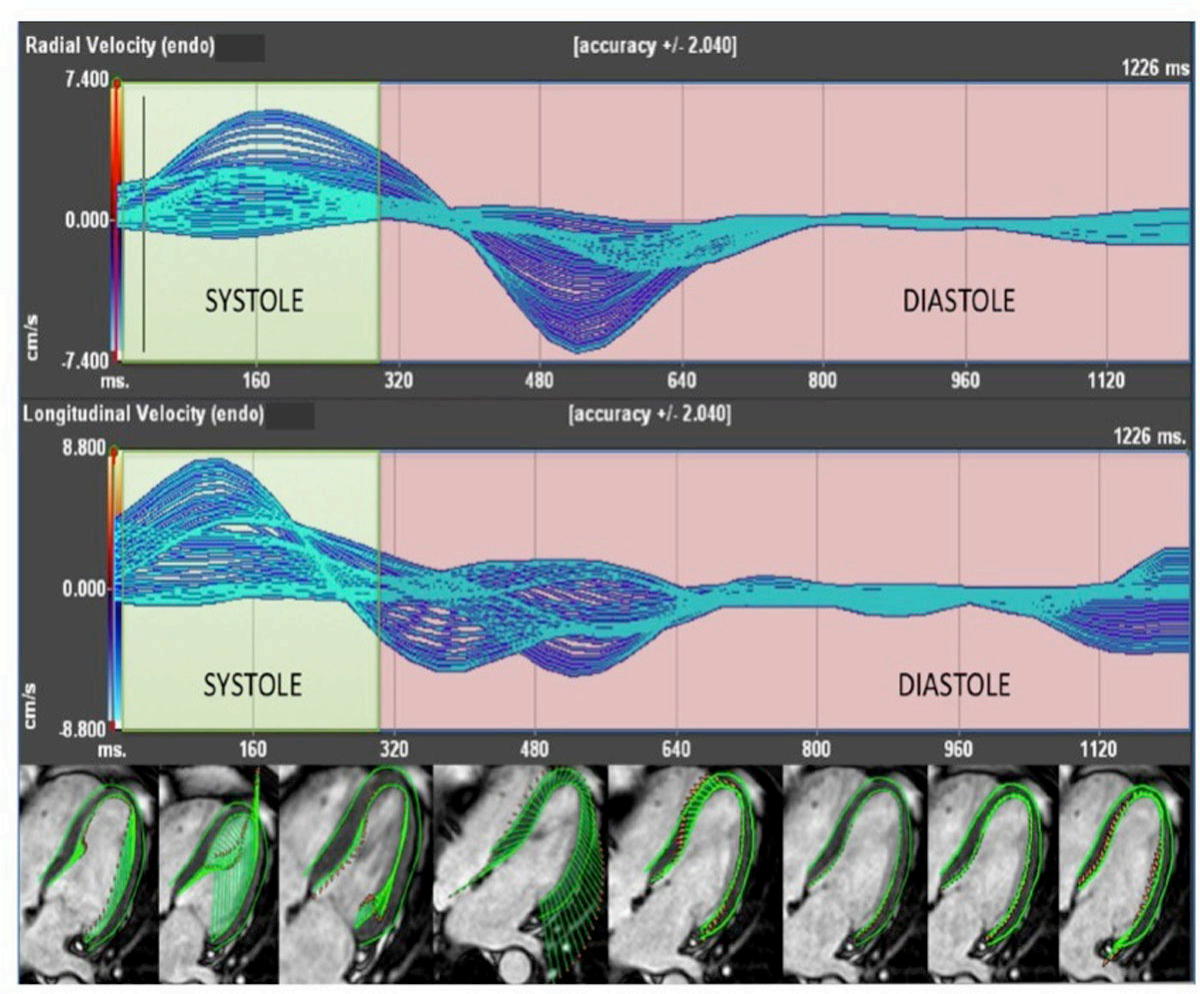
Feature tracking analysis of left ventricle in 4-chamber view.

SAa peak radial strain rate and 4c peak longitudinal velocity showed significant interval change, both reduced between baseline and follow-up (1.72 ± 0.44 vs 1.42 ± 0.30 p= 0.007 and 2.87 ±1.08 vs 2.19 ± 0.84 p=0.006 respectively). Baseline 4c peak longitudinal velocity (figure 2) appeared to be independently associated with EF change using linear regression analysis with higher velocities predicting improvement in EF over time (β= 0.574, p=0.016).

## Conclusions

FT is a sensitive technique to document change in cardiac function when ejection fraction is normal or unchanged. In addition, FT parameters have the potential to predict change in cardiac function. Further studies assessing FT in myocarditis are required to assess potential for improving outcome prediction in myocarditis.

## Funding

MK has received research funding from Barts Charity.

